# Teratoma With Malignant Ectomesenchymoma in the Pineal Region: A Case Report

**DOI:** 10.7759/cureus.27711

**Published:** 2022-08-05

**Authors:** Rebeca Hernández-Reséndiz, Eliezer Villanueva-Castro, Laura Chávez-Macías, Erick Gómez-Apo, Alma Ortiz-Plata, Citlaltepetl Salinas-Lara, Carlos Peñafiel-Salgado, Martha Lilia L Tena-Suck

**Affiliations:** 1 Pediatric Neurology, Hospital Angeles Universidad, Mexico City, MEX; 2 Department of Neurosurgery, Instituto Nacional de Neurología y Neurocirugía Manuel Velasco Suárez, Mexico City, MEX; 3 Department of Pathology, Hospital General de México Dr. Eduardo Liceaga, Mexico City, MEX; 4 Department of Neuropathology, Instituto Nacional de Neurología y Neurocirugía Manuel Velasco Suárez, Mexico City, MEX

**Keywords:** malignant ectomesenchymoma, rhabdomyosarcoma, astrocytoma, pineal region tumor, malignant transformation, teratoma, germinoma

## Abstract

Tumors involving the pineal gland include germinomas, non-germinomatous, and parenchymal tumors. Sometimes these tumors can be differentiated into rhabdomyosarcoma, which is an aggressive and rapidly recurring sarcoma but is a rare event. We present the case of a 23-year-old male, with an eight-year-long history of a non-treated brain tumor compatible with a teratoma. Chemotherapy and radiotherapy were offered, and two years later, malignant transformation to astrocytoma, rhabdomyosarcoma, neural cell carcinoma, ganglioglioma, and low-grade chondrosarcoma was noted. Immunohistochemistry was valuable in differentiating these entities that confirmed the diagnosis. Malignant transformations may be secondary to the normal transformation of multipotent embryonic cells into more developed tissues after radiotherapy of teratoma and malignant ectomesenchymoma transformation.

## Introduction

Pineal region tumors (PRT) are rare compared to other brain neoplasms. These tumors represent fewer than 0.4-1% of central nervous system (CNS) tumors and are more frequent in male children, with a median age at presentation of 12-13 years [1]. Depending on its type, size, and localization, they can cause an intracranial mass effect with motor abnormalities, visual disturbances, aqueductal stenosis with hydrocephalus, and intracranial hypertension symptoms, or even diabetes insipidus and slow growth associated with compressive hypothalamic syndromes [1,2].

Germ cell tumors are the most common of the PRTs, accounting for 50% of cases. These neoplasms are usually divided into germinomas, nongerminomatous germ cell tumors, and parenchymal tumors [2]. Teratomas are included in the second division and comprise 2-4% of intracranial tumors in patients aged less than 15 years [3]. It is known that malignant teratomas occur in 3-6% of patients with metastatic germ cell tumors treated with chemotherapy. They reveal several histological patterns that may include carcinoma, various types of sarcomas, and rhabdomyosarcomatous elements which have more risk of progression and chemoresistance [4].

Malignant ectomesenchymoma (MEM) is an uncommon sarcoma. It has mesenchymal and neuroectodermal elements and originates from pluripotent embryologic migratory neural crest cells [5]. To our knowledge, there are only two previously reported cases of this tumor in the pineal region [6,7].

Here, we report a case with an intracranial germinoma that developed into a teratoma with malignant transformation with embryonal carcinoma, chondrosarcoma, astrocytoma, and rhabdomyosarcoma components, indicating the importance of initial radiation therapy for intracranial germinomas. The coexistence of these varied components in the same tumor is termed MEM.

## Case presentation

**Figure 1:**
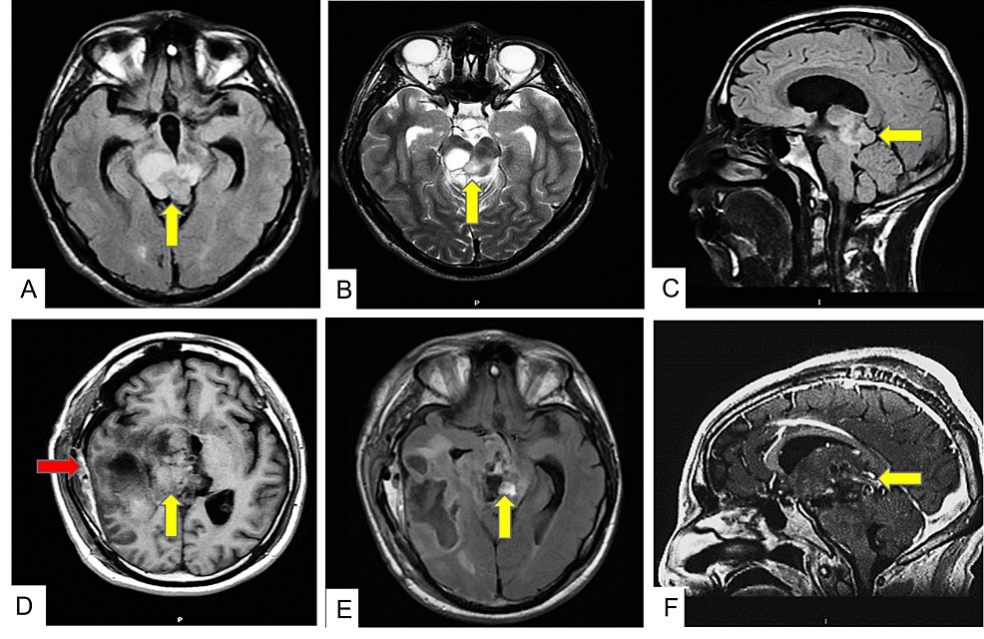
Brain magnetic resonance imaging. First MRI with pineal region tumor. (A) Hyperintense signal in the pineal region in axial T2-FLAIR (yellow arrow). (B) Multiloculated in axial-T2 (yellow arrow). (C) Sagittal-T2-FLAIR (yellow arrow). (D) Second MRI with post-surgical changes (red arrow), tumor growth in axial T1 (yellow arrow). (E) Hyperintense signal in axial T2-FLAIR (yellow arrow). (F) Sagittal T1 with gadolinium enhancement (yellow arrow). MRI: magnetic resonance imaging; FLAIR: fluid-attenuated inversion recovery

**Figure 2:**
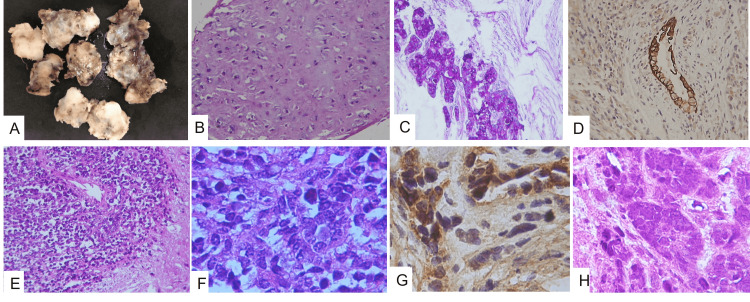
Gross and microscopic features of malignant ectomesenchymoma tissue. (A) Gross aspects of the tumor showing multinodular heterogeneity, white-yellowish, solid myxoid, soft, and cyst formations. (B) Light microscopic examination showing mature fat and chondroid cartilage with focal atypia (H&E ×20). (C) Glandular tissue PAS positive (PAS stain ×20). (D) Other glandular structures with intestinal appearance were also observed to be positive for keratin 20 (IHC ×20). (E) Sheets of primitive tumors with small and hyperchromatic cells with neuroblastic pseudorosettes (H&E ×200). (G) Small hyperchromatic neoplastic cells with neuroepithelial appearance (H&E ×40). (F) Cells with CD56 immunoreaction (IHC stain ×40). (H) glandular epithelial cells with dark staining, large nuclei, increased nucleus-to-cytoplasm ratio, mitosis, and nucleoli in tubular arrangements consistent with adenocarcinoma (H&E ×40). H&E: hematoxylin and eosin; PAS: periodic acid-Schiff; IHC: immunohistochemical

**Figure 3:**
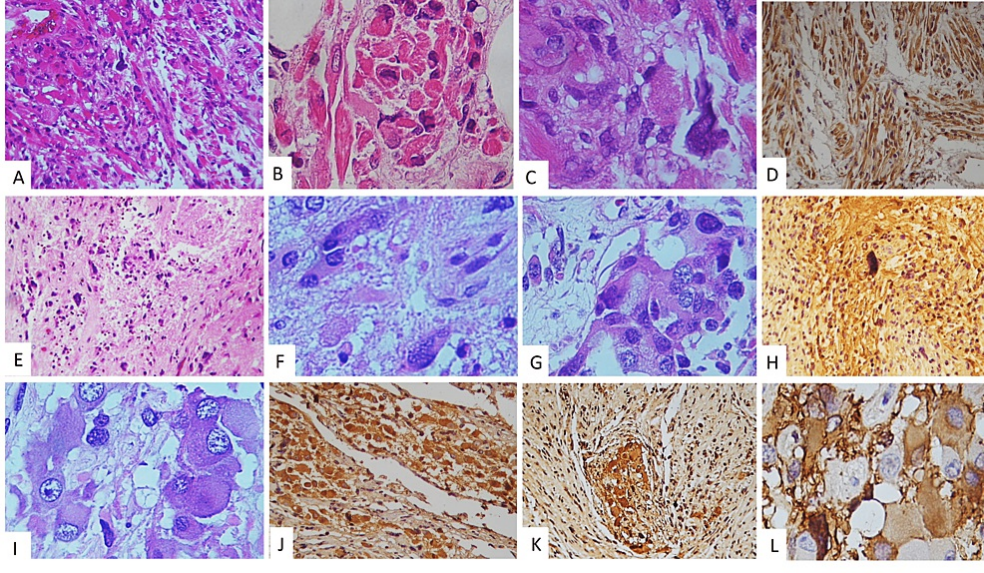
Light microscopic examination demonstrating other histological patterns. (A) Differentiated spindle cells with eosinophilic cytoplasm that match with striated muscle cells (H&E ×20). (B) Giant eosinophilic and rhabdoid cells can be observed (H&E ×40). (C) Cells showing intracellular vacuole and pleomorphic nuclei (H&E ×40). (D) Cells with myogenic positive immunoreaction (IHC stain ×200). (E) Astrocytic differentiation (H&E ×20). (F) Elevated nuclear-to-cytoplasmic ratio and pleomorphic atypical cells consistent with anaplastic astrocytoma. (G) Pleomorphic astrocytes can be observed (H&E ×400). (H) Immunohistochemical staining of the tissue with GFAP showing a positive reaction. (I) Other types of large cells with abundant atypical eosinophilic cytoplasm but with prominent nucleoli suggesting ganglion cells (H&E ×40). (J) Neu-N-positive cells. (K) Positive synaptophysin immunoreaction. (L) GFAP negative (original magnification ×400). H&E: hematoxylin and eosin; IHC: immunohistochemical; Neu-N: neuronal nuclear antigen; GFAP: glial fibrillary acidic protein

This is the case of a 23-year-old male with a history of a pineal germ cell tumor at age 10 who did not receive any treatment until eight years later with radiotherapy and chemotherapy. Two years later, he was admitted to our institution at the Emergency Room Department because of a severe headache and vomiting episodes. Soon, he became lethargic with motor weakness in both legs and complete Parinaud syndrome with paralysis of upward conjugate gaze. Neuroimaging revealed a third ventricle obstructive hydrocephalus caused by a heterogeneous mass originating in the epithalamic region. The patient had a previous magnetic resonance imaging (MRI) with a hyperintense signal multiloculated tumor in the pineal region (Figures 1A-1C). Serum α-fetoprotein (AFP) showed levels above 2.9 × 10^6^ UI/L. The tumor was completely removed with a right subtemporal craniotomy. The post-surgical MRI showed tumor growth and gadolinium enhancement (Figures 1D-1F). The tumor was histologically diagnosed as immature teratoma with malignant transformation. It was a large and well-circumscribed mass with multifocal hemorrhages and cystic changes in the cut surface with a myxoid aspect and yellowish white soft tissue that measured 6 × 6 cm (Figure 2A). Histopathology revealed that the resected mass had a composition based on mature adipose tissue, chondroid cartilage nests with some atypical cells (Figure 2B), and normal glandular tissue of intestinal and sweat glands (Figure 2C). There were areas with atypia of the glandular epithelium, which showed large hyperchromatic nuclei with dense chromatin, nucleoli, increased nucleus-to-cytoplasm ratio, and mitotic figures in tubular arrangements with pseudorosette formation compatible with carcinoma (Figures 2E-2H). It showed positive immunoreactivity to the following markers: CK6/7/8/20 (Figure 2D), synaptophysin, CD56, CD117, neuronal nuclear antigen (NeuN), AFP, carcinoembryonic antigen (CEA), glial fibrillary acidic protein (GFAP) (Figure 2F), octamer-binding transcription factor 4 (OCT-4), CD99, nuclear transcription factor related to the development of Schwann cells and melanocytes (SOX10), and weak immunoexpression of beta-human chorionic gonadotropin (β-hCG), as well as negativity for a tumor suppressor gene named INI1. Other areas with spindle cells had large cytoplasm mixed with giant cells with numerous nuclei, and some of them showed marked nucleoli (Figure 3, Panel A). Intermixed cells with multinucleated giant cells and large eosinophilic cytoplasm similar to rhabdomyoblasts were also seen (Figure 3, Panel B). Brisk mitotic activity was noted (Figure 3, Panel C). Those cells showed a positive immunoreaction to calcium-binding protein B S-100B, myoglobin, myogenin, and actin (Figure 3, Panel D), but germ cell tumor markers such as creatine kinase (CK), AFP, placental alkaline phosphatase (PLAP), CEA, OCT-4, and were negative.

Another histological pattern showed myxoid areas with focal necrosis in a fibrillary background with large and elongated cells with prominent and atypical pleomorphic nuclei, which gave the appearance of gemistocytic astrocytes alternating with wide eosinophilic cytoplasm cells that had prominent nucleoli and suggested ganglionic neurons (Figure 3, Panels E-G). Giant cells were GFAP positive (Figure 3, Panel H). Ganglionar cells were also observed (Figure 3, Panel I), and those were Neu-N (Figure 3, Panel J), synaptophysin, neuron-specific enolase (NSE), and S-100 positive (Figure 3, Panel K), but negative to GFAP (Figure 3, Panel L). With these heterogeneous histological findings of a mature teratoma with malignant transformation with pattern of embryonic carcinoma, anaplastic astrocytoma, ganglioglioma, low-grade chondrosarcoma, and rhabdomyosarcoma, the final diagnosis was a mixed malignant tumor with many cellular components which fit into the group of tumors named MEM in the pineal region. Postoperatively and during recovery, the patient coursed from reversible impairment of consciousness and polyuria that was treated in a timely manner with progressive improvement. No further postoperative adjuvant therapy was provided.

## Discussion

Sudden and marked elevation of AFP has a strong correlation with malignant transformation or germinoma invasion [3]. Intracranial recurrence of a germinoma can transform into a teratoma, choriocarcinoma, yolk sac tumor, astrocytoma, rhabdoid choroid plexus tumor, and rhabdomyosarcoma [4,8]. The hypothesis suggests that all germ cell tumors originate from primordial germ cells that migrated from the midline during development. Epigenetic and biological alterations such as the mutation on the activation of cascades which have important effects on proliferative, apoptotic, and differentiation pathways, such as the Akt/ mTOR and Kit-, Ras/Raf/Erk-, chromosomal instability, demethylation, as well as gain of chromosomes 12p or X, appear to play a transcendent role in the tumorigenic process and their difference with germinoma origins [9-11].

We present a rare case of pineal germinoma treated with chemotherapy and radiotherapy. After two years of non-symptomatic post-treatment survival, the patient presented with a new tumor that was considered to be a germinoma recurrence; however, due to the cystic component on MRI and the elevation of AFP and β-GCH, a possible teratoma was suggested. The tumor presented several heterogeneous histological patterns as epithelial glands, adipose tissue, muscle, and nerves. These are the morphological features of a classical mature teratoma. However, we also noted small hyperchromatic cells forming pseudoglandular structures and rosettes (neuroepithelial cells), components of mature and immature brain tissue that resembled astrocytoma, atypical pleomorphic and ganglionar neurons with rhabdoid appearance, malignant sarcomatoid neoplasm with rhabdomyoblastic differentiation, and nests of cartilage with atypical cells that are characteristic of low-grade chondrosarcoma. Due to the complexity of the histological patterns, it was finally diagnosed as MEM.

The spindle cells with a rhabdoid appearance showed positive immunoreaction to desmin, myogenin, myoglobin, and actin, and weak immune reaction to CEA and OCT-4, which is characteristic of muscle transformation. However, anaplastic cells and mitotic figures are most indicative of malignant embryonal rhabdomyosarcoma transformation.

Striations and strap cells are only seen in CNS rhabdomyosarcoma on rare occasions, and the majority show globoid cells with eccentric nuclei, elongated strap cells, and undifferentiated small cells consistent with rhabdomyoblasts [12]. In our case, we also found multinucleated myotube-like structures with myogenin, myogenic differentiation 1 factor (MyoD1), focal, weak synaptophysin, NSE, GFAP, CD117, PLAP, CK, and OCT-4.

Seven cases of primary pineal rhabdomyosarcoma have been reported so far in two reviews by Pandey et al. in 2020 and Xie et al. in 2022 [13,14]. In these tumors, light microscopy reveals a tumor with high cellularity with small round cells mixed with others with a large eosinophilic cytoplasm, compatible with multinucleated giant cells and rhabdomyoblasts. The tumor cells that are usually positive for desmin while negative for synaptophysin, GFAP, myogenin, and myogenic differentiation 2 factor (MyoD2) confirm the diagnosis of rhabdomyosarcoma. INI1 was positive as well. All germ cell tumor markers were negative, and the differential diagnosis was with teratoid/rhabdoid tumors and gangliogliomas [15]. INI1 immunostaining, a gene also named *SMARCB1*, is a specific and sensitive test for the diagnosis of atypical teratoid/rhabdoid tumors which have loss of their nuclear expression. In our case, the giant cells with nucleolus that resembled ganglion cells were observed in a fibrillary GFAP-positive stroma. Neurons with immature phenotypes were also identified, and these are not usually found in immature teratoma.

The neuroectodermal component can vary from immature primitive neural elements to clustered ganglion cells forming pseudorosettes [12]. In our case, we reported a giant cell that corresponded to true ganglion cells. However, synaptophysin, GFAP, and CD56 positive staining suggest the presence of neural elements of ectomesenchymoma and confirm the diagnosis [15]. MEMs are believed to originate from pluripotent embryonal migratory neural crest cells able to form neuroectodermal and mesenchymal tissues, but chondrosarcoma, pleomorphic and undifferentiated sarcomas, rhabdomyosarcoma, gliosarcoma, and liposarcoma have also been reported [6,7,15].

β-HCG levels in germinomas are inconsistent, but when found, if elevated, may have a poor prognosis. Markers of germ cell tumors, such as CEA, PLAP, CKs, OCT-4, and CD117 were positive [16]. The AFP levels, MRI findings of a heterogeneous image compatible with cysts, and the loculated form of immature teratoma may be secondary to the normal transformation of multipotent embryonic cells into a mature multinodular appearance as well as malignant transformation. Although specific MRI findings are helpful in the differential diagnosis of pineal tumors, malignant transformation due to the pluripotent ability of germ cell tumor components may occur.

Sarcoma is the most common histological subtype of malignant transformation, followed by adenocarcinoma and primitive neuroectodermal tumor transformation [15].

It is supposed to represent an acceleration of localized dysplastic processes of totipotent germ cells present in the midline neuraxis or the growth of unidentified microscopic residue of the germinoma component in mature teratoma.

## Conclusions

Due to its rarity, distinct nature, remarkable heterogeneity, and diversity of histological malignant patterns, this disease needs to be fully and timely characterized. Germ cell tumors in the pineal region can undergo malignant transformation following standard treatments. We know that the prognosis of a tumor malignant transformation is unfavorable because they tend to recur and often do not respond to chemotherapy and radiotherapy. The progression of this tumor may correlate with overall survival and clinical outcomes but there are too few cases reported so far and more studies are needed.
